# β-Cell-Specific Glucocorticoid Reactivation Attenuates Inflammatory β-Cell Destruction

**DOI:** 10.3389/fendo.2014.00165

**Published:** 2014-10-14

**Authors:** Xiaoxia Liu, Sophie Turban, Roderick N. Carter, Shakil Ahmad, Lynne Ramage, Scott P. Webster, Brian R. Walker, Jonathan R. Seckl, Nicholas M. Morton

**Affiliations:** ^1^Molecular Metabolism Group, University/BHF Centre for Cardiovascular Science, Queen’s Medical Research Institute, Edinburgh, UK; ^2^Division of Endocrinology and Metabolism, Department of Internal Medicine, Huashan Hospital, Shanghai Medical College, Fudan University, Shanghai, China; ^3^Aston Medical School, Aston University, Birmingham, UK; ^4^Endocrinology Unit, University/BHF Centre for Cardiovascular Science, Queen’s Medical Research Institute, Edinburgh, UK

**Keywords:** glucocorticoids, 11beta-hydrosteroid dehydrogenase type 1, type 1 diabetes, inflammation, beta-cells, anti-inflammatory agents, insulin secretion, streptozotocin

## Abstract

Progression and severity of type 1 diabetes is dependent upon inflammatory induction of nitric oxide production and consequent pancreatic β-cell damage. Glucocorticoids (GCs) are highly effective anti-inflammatory agents but have been precluded in type 1 diabetes and in islet transplantation protocols because they exacerbated insulin resistance and suppressed β-cell insulin secretion at the high-doses employed clinically. In contrast, physiological-range elevation of GC action within β-cells ameliorated lipotoxic β-cell failure in transgenic mice overexpressing the intracellular enzyme 11β-hydroxysteroid dehydrogenase type 1 (MIP-HSD1^tg/+^ mice). Here, we tested the hypothesis that elevated β-cell 11beta-HSD1 protects against the β-cell destruction elicited by streptozotocin (STZ), a toxin that dose-dependently mimics aspects of inflammatory and autoimmune β-cell destruction. MIP-HSD1^tg/+^ mice exhibited an episodic protection from the severe hyperglycemia caused by a single high dose of STZ associated with higher and sustained β-cell survival, maintained β-cell replicative potential, higher plasma and islet insulin levels, reduced inflammatory macrophage infiltration and increased anti-inflammatory T regulatory cell content. MIP-HSD1^tg/+^ mice also completely resisted mild hyperglycemia and insulitis induced by multiple low-dose STZ administration. *In vitro*, MIP-HSD1^tg/+^ islets exhibited attenuated STZ-induced nitric oxide production, an effect reversed with a specific 11beta-HSD1 inhibitor. GC regeneration selectively within β-cells protects against inflammatory β-cell destruction, suggesting therapeutic targeting of 11beta-HSD1 may ameliorate processes that exacerbate type 1 diabetes and that hinder islet transplantation.

## Introduction

Type 1 diabetes is a chronic disease characterized by inflammatory β-cell destruction secondary to an initial autoimmune targeting of the islets (1). Inflammatory macrophages are key to the development and maintenance of islet damage (2). Pro-inflammatory cytokines derived from macrophages and damaged β-cells further suppress β-cell function in part through induction of nitric oxide production (3, 4). As type 1 diabetes progresses, pro-inflammatory cytokines inhibit β-cell regeneration, stimulate peripheral insulin resistance and maintain insulitis (1).

Glucocorticoids (GCs) are used clinically due to their potent anti-inflammatory and immunosuppressive effects (5) but were excluded as a treatment for type 1 diabetes and in transplant protocols (6) because they promoted peripheral insulin resistance and suppressed β-cell function at the high-doses employed (7–9). However, the prevailing dogma that GC action on β-cells is purely deleterious has been increasingly challenged (10–14). Exposure of normal mouse islets to GCs can improve aspects of secretory function through suppression of inflammatory signaling (10). Moreover, pre-treatment of islets with GCs (11) or localized exposure of transplanted islets to GCs contained within their surrounding implant matrix (12) has shown improved efficacy and graft survival. Crucially, transgenic mice with modest β-cell-specific elevation of the intracellular GC regenerating enzyme 11β-hydroxysteroid dehydrogenase (HSD11b1; 11beta-HSD1; MIP-HSD1 mice) exhibited protection from lipotoxic β-cell failure *in vivo* as a result of increased islet number, arising from a post-developmental effect, and function, due to enhanced secretory capacity and cell survival signaling (14).

The beneficial effects of β-cell-specific 11beta-HSD1 elevation (14) were manifest in a chronic high-fat feeding obesity model. Although obesity is associated with a low-grade inflammation of the islets (15), the protective mechanisms found in MIP-HSD1 islets were not obviously anti-inflammatory (14). Therefore, the impact of intra-β-cell GC regeneration on the processes of cellular damage occurring in profoundly inflammatory contexts relevant to type 1 diabetes remains unknown. To address this we tested the hypothesis that elevated β-cell 11beta-HSD1 protects against the profound β-cell destruction or inflammatory insulitis driven by distinct doses of the β-cell toxin streptozotocin (STZ).

## Materials and Methods

### Animals

All experiments conformed to local ethical guidelines of the University of Edinburgh and the UK Home Office Animals (Scientific Procedures) Act (1986). Male MIP-HSD1^tg/+^ and C57BLKS/J (KsJ) littermate control mice (14) were housed in standard conditions on a 12 h light/dark cycle and fed standard rodent chow (Special Diet Services, Edinburgh, UK). Age matched 10–12-week-old male mice were used for all the experiments.

### Streptozotocin treatments

Mice were injected intraperitoneally with a single bolus of STZ (180 mg/kg/body weight) or for five consecutive days with 40 mg/kg/body weight STZ dissolved in 10 mmol/l sodium citrate (pH 4.5) or vehicle. Blood glucose was measured (OneTouch Ultra, Johnson and Johnson, Bucks, UK) from a tail venesection. Mice were sacrificed at 3 and 10 days (single dose) or 15 days (multiple dose) after injection. Insulin was measured by ELISA (Crystal Chem, Downers Grove, IL, USA).

### Immunohistochemistry

Pancreata were fixed in 4% paraformaldehyde, paraffin embedded, sectioned (4 μm), and immunostained with guinea pig anti-insulin (1:300) (AbCam, Cambridge, UK), rabbit anti-Mac-2 (1:150) (Cedarline, ON, Canada), rabbit anti-FOXP3 (1:150) (eBioscence, Hatfield, UK), rabbit anti-NEUROG3 (1:1000), and rabbit anti-SOX9 (1:8000) (Millipore Corporation, Bellirica, MA, USA). For chromogen labeling with diaminobenzidine (DAB) (Dakocytomation, Carpinteria, CA, USA) biotinylated anti-guinea pig and anti-rabbit (AbCam) secondary antibodies were used. Image and quantification of positive cells in islet areas were carried out using KS300 software (3.0 CarlZeiss Vision, GmBH) or computerized image analysis (MCID Basic 7.0 software) for analysis of the whole sections. For immunofluorescence, sections were incubated with rabbit anti-ki67 (1:3000, Dakocytomation) then goat anti-rabbit peroxidase (Abcam) followed by Tyramide green 488 (Perkin Elmer, Cambridge, UK) then incubated with rabbit anti-PDX1 (1:1000, Millipore). After antigen retrieval, sections were incubated with goat anti-rabbit Alexa Fluor 546 (1:200, Molecular probes, Paisley, UK) and DAPI (1:1000, Sigma Aldrich, Dorset, UK) and visualized using a Leica fluorescence microscope. Quantification for PDX1 and Ki67 was performed using Image J software (http://www.ncbi.nlm.nih.gov).

### Islet isolation and preparation

Pancreata were digested with collagenase XI (Sigma Aldrich) and islets were hand-picked under a stereomicroscope in Hank’s Balanced Salt Solution, 10% FBS (Lonza, Berkshire, UK). Batches of 80 islets were incubated in RPMI-1640 (Gibco, Life Technologies, Paisley, UK), 10% FBS, 6.1 mmol/l d-glucose, 2 nmol/l 11-dehydrocorticosterone with or without 10 mmol/l STZ diluted in sodium citrate 10 mmol/l and with or without L-NAME (Sigma) 5 mmol/l for 72 h on 8 μm inserts (Millipore). Pictures of the islets were taken using a Zeiss microscope and media were collected for measurement of nitric oxide.

### Nitric oxide (NO) production

Total NO in the media was assayed as nitrite, the stable breakdown product of NO, using a Sievers chemiluminescence analyzer (Analytix, Sunderland, UK). Islets were homogenized in lysis buffer as described in (14) and protein content evaluated by Biorad assay (BioRad Laboratories, Hercules, CA, USA).

### Statistics

Data are expressed as mean ± SEM and were analyzed using one-way ANOVA (Newman–Keuls *post hoc* test).

## Results

### MIP-HSD1^tg/+^ mice resist high-dose STZ-induced hyperglycemia

**Figure 1:**
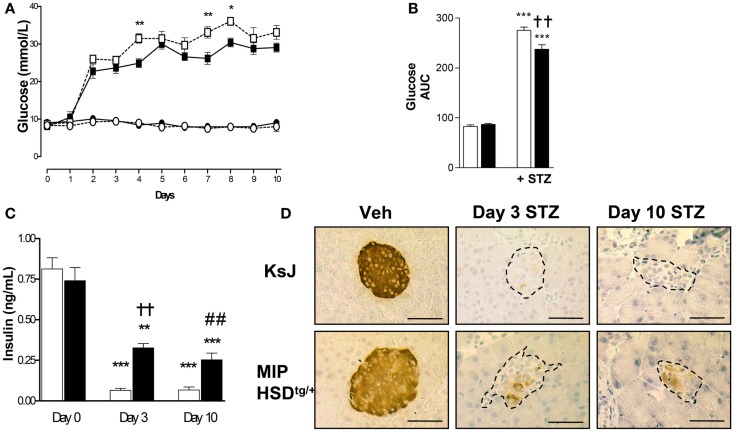
**MIP-HSD1^tg/+^ mice exhibit episodic improvement in hyperglycemia and higher islet insulin production after high-dose STZ treatment**. Twelve-week-old mice were given a single i.p. injection of STZ (180 mg/kg/bodyweight) or citrate buffer as vehicle. **(A)** Daily blood glucose in littermate vehicle-injected KsJ mice (●, *n* = 5) and MIP-HSD1^tg/+^ mice (∘, *n* = 7) or after STZ injection in KsJ mice (□, *n* = 8) and MIP-HSD1^tg/+^ mice (■, *n* = 12). Values represent mean ± SEM, differences were analyzed by one-way ANOVA, Newman–Keuls test, *P < 0.05 and **P < 0.01 for MIP-HSD1^tg/+^ STZ vs. KsJ STZ. **(B)** Area under the curve of overall blood glucose levels in littermate KsJ mice (white bars) and MIP-HSD1^tg/+^ mice (black bars). ***P < 0.001 for STZ vs. vehicle, **P < 0.01 for MIP-HSD1^tg/+^ STZ vs. KsJ STZ. **(C)** Plasma insulin at day 0 (before injection) and days 3 and 10 post-STZ injection in littermate KsJ mice (white bars) and MIP-HSD1^tg/+^ mice (black bars). **P < 0.01 and ***P < 0.001 day 3 and 10 vs. day 0. The decrease in plasma insulin was less severe in transgenic mice, ^††^*P* < 0.01 for day 3 MIP-HSD1^tg/+^ STZ vs. day 3 KsJ STZ; ^##^*P* < 0.01 for day 10 MIP-HSD1^tg/+^ STZ vs. day 10 KsJ STZ. **(D)** Insulin expression shown by immunohistochemical staining of paraffin-fixed pancreata from KsJ littermate (upper row) and MIP-HSD1^tg/+^ (lower row) mice after vehicle treatment (Veh, left lane) and 3 (middle lane) or 10 days post-STZ treatment (right lane). Bars = 50 μm magnification ×400.

We began by administering a high-dose of STZ (180 mg/kg body weight) known to completely ablate β-cell function (16). High-dose STZ caused marked and comparable hyperglycemia by 2 days in MIP-HSD1^tg/+^ and control non-transgenic littermates (KsJ), indicating comparable ablation of β-cell function. However, after day 3, during the inflammatory-response phase of islet destruction, MIP-HSD1^tg/+^ mice began to exhibit episodic phases of significantly less severe hyperglycemia than KsJ mice (Figures 1A,B) suggestive of partial recovery of function in existing β-cells and/or spontaneous regeneration of new β-cells. Plasma insulin levels were significantly higher in MIP-HSD1^tg/+^ than in KsJ mice at day 3 and 10 (Figure 1C), consistent with their residual islet insulin staining and further supporting an islet-specific β-cell recovery (Figure 1D). Notably, circulating corticosterone levels were markedly elevated by high STZ, but to a similar degree in KsJ and MIP-HSD1^tg/+^ mice (nmol/l: KsJ vehicle: 131 ± 28, KsJ high STZ: 535 ± 155, MIP-HSD1^tg/+^ vehicle: 138 ± 45 MIP-HSD1^tg/+^ high STZ: 683 ± 125, no significant effect of genotype), supporting a role for local β-cell GC regeneration as the underlying driver of genotype-specific effects.

### MIP-HSD1^tg/+^ mice maintain higher β-cell mass and replicative capacity after STZ

**Figure 2:**
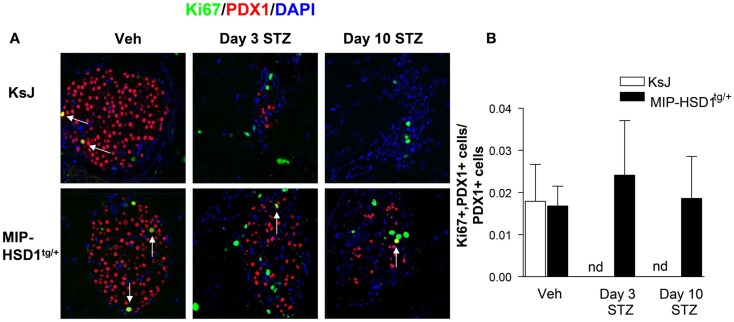
**MIP-HSD1^tg/+^ mice exhibit enhanced β-cell survival and maintained replicative capacity after STZ treatment**. **(A)** Representative immunofluorescence images of paraffin-fixed pancreata co-stained with rabbit anti-Ki67 (green), rabbit anti-PDX1 (red), and DAPI (blue) from KsJ littermate (upper row), MIP-HSD1^tg/+^ (lower row) mice after vehicle treatment (Veh, left lane), 3 (middle lane) and 10 days post-STZ treatment (right lane). Arrows indicate double-positive stained cells, magnification ×400. **(B)** PDX1 and Ki67 positive cells were counted within the islets as defined morphologically by the islet capsule boundary using Image J software. Proliferating β-cells were measured as a ratio of PDX1, Ki67 double-positive cells over total PDX1-positive cells. Ki67 and PDX1 double-positive staining was undetectable (nd) in KsJ mice 3 and 10 days post-STZ treatment. Values represented mean ± SEM, differences were analyzed by one-way ANOVA, Newman–Keuls test (*n* = 6–12).

The remarkable resilience of MIP-HSD1^tg/+^ β-cells against high-dose STZ could be due to higher β-cell survival or increased spontaneous β-cell regeneration. Of note, there was low and comparable β-cell proliferation (~2% of total islet cell number double-positive for the proliferation marker Ki67 and the β-cell marker PDX1) in vehicle-treated KsJ and MIP-HSD1^tg/+^ mice (Figure 2A, left lane and quantification Figure 2B). Islet PDX1-positive β-cell number was severely reduced by day 3 and continued to fall by day 10 to undetectable levels in KsJ mice treated with STZ. Replicating Ki67 single-positive cells in islets of STZ-treated KsJ mice are likely infiltrating immune cells. Ki67/PDX1 double-positive cells were undetectable in KsJ islets indicating a complete loss of β-cell replicative capacity (Figure 2A, upper row, middle, and right lane and quantification, Figure 2B). Despite a marked reduction in PDX1-positive cells, a substantial β-cell number remained in STZ-treated MIP-HSD1^tg/+^ islets, although the comparable hyperglycemia between genotypes from day 2 to 3 indicates these β-cells underwent a period of initial secretory dysfunction (Figure 1). MIP-HSD1^tg/+^ mice also maintained their Ki67/PDX1 double-positive cell number in their remaining islets (Figure 2A bottom row and quantification Figure 2B) suggesting that their β-cell replicative capacity is maintained after STZ. This could not be accounted for by β-cell neogenesis from potential progenitor cell types, as assessed with SOX9 and NEUROG3 (17, 18) immunostaining that did not differ by genotype 3 or 10 days after STZ (Figures S1 and S2 in Supplementary Material). Apoptosis was not detected in islets of either genotype by a number of methods suggesting that this process does not account for the differences between the genotypes under these experimental conditions (Figure S3 in Supplementary Material).

### MIP-HSD1^tg/+^ islets have reduced macrophage and increased T regulatory cell infiltration after STZ

**Figure 3:**
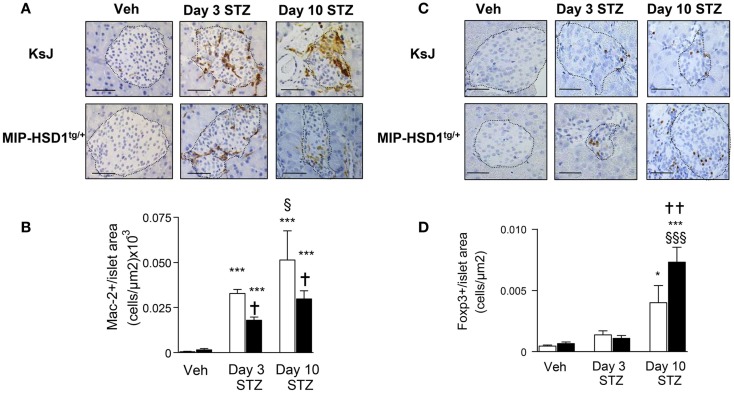
**MIP-HSD1^tg/+^ mice have reduced islet macrophage infiltration and increased T regulatory cell numbers after STZ administration**. **(A)** Immunohistochemical staining for Mac-2 with **(B)** quantification and **(C)** FOXP3 immunohistochemical staining with **(D)** quantification. **(A,C)** Pancreata from KsJ littermate (upper rows), MIP-HSD1^tg/+^ (lower rows) mice after vehicle (left lane) or 3 (middle lane) and 10 days (right lane) post-STZ treatment were paraffin-fixed and sectioned. **(C,D)** Mac-2 and FOXP3 positive cells per islet area were quantified using Zeiss (KS300 3.0) software. Values represent mean ± SEM. Differences were analyzed by one-way ANOVA and Newman–Keuls test, *P < 0.05 and ***P < 0.001 for STZ vs. Veh; *P < 0.05 and **P < 0.01 for MIP-HSD1^tg/+^ vs. KsJ control; ^§^*P* < 0.05 and ^§§§^*P* < 0.001 STZ D10 vs. STZ D3 (*n* = 6–10). Bars = 50 μm, magnification ×400.

Inflammatory macrophage infiltration is an early event in autoimmune (2) and STZ-induced islet damage (19). MIP-HSD1^tg/+^ islets had fewer (~40%) infiltrated macrophages, as assessed by the macrophage marker Mac-2, compared to KsJ controls at 3 and 10 days after the single high-dose STZ injection (Figure 3A and quantification, Figure 3B).

T regulatory cells (Treg; FOXP3^+^ cells) mediate inflammatory resolution and retard the progression of diabetes (20). Treg cell numbers increased modestly (twofold) in both normal KsJ and MIP-HSD1^tg/+^ islets/peri-islet area 3 days post-STZ. Treg cell numbers continued to increase significantly only in MIP-HSD1^tg/+^ islets by day 10 post-STZ (Figure 3C, and quantification Figure 3D).

### MIP-HSD1^tg/+^ mice resist multiple low-dose STZ-induced hyperglycemia

**Figure 4:**
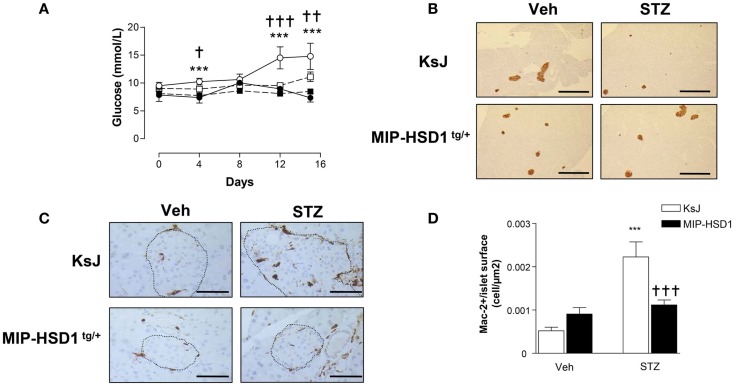
**MIP-HSD1^tg/+^ mice completely resist hyperglycemia induced by multiple low-dose injection of STZ**. Twelve-week-old mice were injected i.p. either with sodium citrate vehicle or STZ (40 mg/kg/body weight) for five consecutive days. **(A)** Blood glucose levels measured immediately before injections (day 0) and day 4, 8, 12, and 15 after vehicle injection in littermate KsJ mice (●, *n* = 5) or MIP-HSD1^tg/+^ mice (■, *n* = 7) and after STZ injection in littermate KsJ mice (∘, *n* = 8) or MIP-HSD1^tg/+^ mice (□, *n* = 12). Blood glucose level in MIP-HSD1^tg/+^ STZ-treated mice was significantly elevated only KsJ STZ-treated mice. Values represented mean ± SEM. Differences were analyzed by one-way ANOVA, Newman–Keuls test, ***P < 0.001 for KsJ STZ vs. KsJ Veh; *P < 0.05 and **P < 0.01, ^†††^*P* ≤ 0.001 for MIP-HSD1^tg/+^ STZ vs. KsJ STZ. **(B)** Preservation of islet structure in MIP-HSD1^tg/+^ shown by insulin staining of paraffin-fixed pancreata from KsJ mice (upper row) and MIP-HSD1^tg/+^ mice (bottom row) 10 days after the end of vehicle (Veh, left lane) or STZ (right lane) treatment. Scale = 400 μm magnification ×50. **(C)** Reduced macrophage infiltration in MIP-HSD1^tg/+^ shown by Mac-2 staining of paraffin-fixed pancreata from KsJ mice (upper row) and MIP-HSD1^tg/+^ mice (bottom row) 10 days after the end of vehicle (Veh, left lane) or STZ (right lane) treatment. Bars = 50 μm, magnification ×400. **(D)** Quantitation of Mac-2 positive cells per islet area using Zeiss (KS300, 3.0) software. Values represent mean ± SEM, differences were analyzed by one-way ANOVA, Newman–Keuls test, ***P < 0.001 for STZ vs. Veh; ***P < 0.001 for MIP-HSD1^tg/+^ STZ vs. KsJ STZ (*n* = 5–10).

To test responses to a more subtle diabetic insult that recapitulates some of the inflammatory and autoimmune aspects of type 1 diabetes (21, 22), a low-dose of STZ (40 mg/kg/BW) was administered for five consecutive days. KsJ mice showed significant hyperglycemia from day 4 that reached a modestly diabetic plateau by day 12 (Figure 4A), had reduced islet insulin staining (Figure 4B) and increased islet macrophage numbers, whereas MIP-HSD1^tg/+^ mice maintained normal glycemia and pancreatic morphology and displayed abrogated macrophage infiltration (Figure 4C, and quantification, Figure 4D).

### β-Cell 11β-HSD1 activity curtails islet STZ-induced nitric oxide production

**Figure 5:**
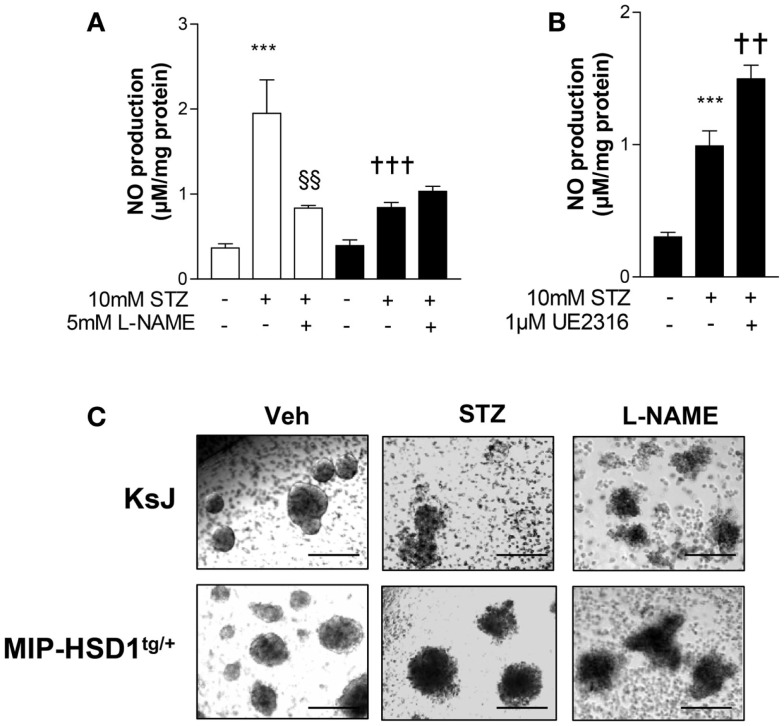
**Isolated MIP-HSD1^tg/+^ islets show attenuated induction of nitric oxide production and resistance to disintegration in culture after STZ challenge**. Islets from 12-week-old mice were isolated and incubated *in vitro* with vehicle or STZ (10 mmol/l) with or without the nitric oxide synthase inhibitor L-NAME (5 mmol/l) in the presence of 2 nmol/l 11-dehydrocorticosterone 11beta-HSD1 substrate for 72 h. **(A)** Analysis of nitric oxide (NO) production in media after 72 h incubation with vehicle (−), STZ or STZ + L-NAME from islets from KsJ (white bars) or MIP-HSD1^tg/+^ mice (black bars). Values represent mean ± SEM. Differences were analyzed by one-way ANOVA, Newman–Keuls test, ***P < 0.001 for STZ vs. Veh; ^†††^*P* < 0.001 for MIP-HSD1^tg/+^ STZ vs. KsJ STZ, ^§§^*P* < 0.01 STZ + L-NAME vs. STZ (*n* = 6). **(B)** NO production in media of isolated islets from MIP-HSD1^tg/+^ mice treated with STZ with or without 11beta-HSD1 inhibitor UE2316 (1 μmol/l) for 72 h. Values represent mean ± SEM, differences analyzed by one-way ANOVA, Newman–Keuls test, ***P < 0.001 for STZ vs. Veh; **P < 0.01 for STZ + UE2316 vs. STZ (*n* = 6). **(C)** Representative light microscopy images of islets from KsJ (upper row) and MIP-HSD1^tg/+^ mice (lower row) incubated with vehicle (left lane), with STZ (middle lane), or with STZ + L-NAME (right lane). Magnification ×400.

Inflammatory mediators induce production of nitric oxide (NO) that causes β-cell destruction and acts as an important chemoattractant for macrophages (1, 3, 4). STZ can induce inflammatory pathways and islet damage in part by generating NO (23). Incubation of normal KsJ islets with STZ stimulated NO production (Figure 5A, white bars) and caused islet disintegration (Figure 5C, middle lane, top row). The STZ-induced rise in NO and islet damage was attenuated by the iNOS inhibitor, L-NAME (Figures 5A,C, right lane). NO production was suppressed after STZ treatment of MIP-HSD1^tg/+^ islets (Figure 5B, black bars) and islet disintegration was attenuated (Figure 5C, middle lane, bottom row). The specific 11beta-HSD1 inhibitor UE2316 (14) reversed suppression of NO found in MIP-HSD1^tg/+^ islets in the presence of the 11beta-HSD1 substrate 11-dehydrocorticosterone, confirming that β-cell 11beta-HSD1 activity inhibits NO production.

## Discussion

Modest elevation of 11beta-HSD1 activity in β-cells (14) has conferred improved β-cell survival and a sustained capacity for spontaneous β-cell regeneration in the context of severe inflammatory β-cell destruction. Although the protection against hyperglycemia is modest across the short-time course of the present studies, the continued survival and replenishment of functional β-cells after high-dose STZ points to a remarkable and unexpectedly effective protective role for local GC regeneration. Hyperglycemia induced by multiple low-dose STZ, a regimen that invokes some of the autoimmune and inflammatory processes (21, 22, 24) to that found in type 1 diabetes, was completely prevented in MIP-HSD1^tg/+^ mice.

The protective effect of elevated β-cell 11beta-HSD1 involves both increased β-cell survival and maintained β-cell replicative potential. Improved MIP-HSD1^tg/+^ islet survival is mediated by GC-mediated suppression of pro-inflammatory NO production (3, 4), an effect of GCs also found in the vasculature (25). Increased expression of heat shock and other cellular stress–resistance pathways in MIP-HSD1^tg/+^ islets (14) is consistent with their augmented survival, and may counteract triggering of more aggressive inflammatory responses after β-cell insult.

Under normal physiological conditions, β-cell mass is maintained through slow rates of renewal and turnover (26). Hyperglycemia can prompt islet mass compensation predominantly through hypertrophy of existing β-cells (27), β-cell proliferation (26), and neogenesis from progenitors, at least in pancreatic injury models (18). However, we found no evidence for altered ductal (SOX9) or islet endocrine (NEUROG3) progenitor cells as the basis of maintained β-cell mass in STZ-treated MIP-HSD1^tg/+^ mice, in agreement with recent findings that β-cell neogenesis does not come from ductal cell progenitors in the adult pancreas (17). Thus, altered GC regeneration selectively impacts upon β-cell replication in the STZ-injured adult pancreas. Moreover, MIP-HSD1^tg/+^ islets exhibit a hyper-functionality associated with increased Cdkn1a (P21) expression suggesting accelerated functional maturation of MIP-HSD1 β-cells (14), consistent with the role of GCs in terminal differentiation. Newly generated MIP-HSD1 β-cells likely achieve functionality more rapidly than normal β-cells. In support of this notion, overexpression of the pro-differentiation factor P21 in β-cells promotes resistance to high-dose STZ by increasing progenitor differentiation (28). Notably, apoptosis was undetectable in STZ-treated KsJ mouse islets and pancreas, despite a strong signal in our positive control spleen tissue (Figure S3B in Supplementary Material). It cannot be ruled out that our time points missed a significant incidence of apoptosis, as noted by others (29) or that rapid turnover of apoptotic cells markers [within minutes; (30)] may have caused the signal to fall below our limit of detection. Experimental designs more suited to inducing β-cell apoptosis will be needed to determine the contribution of altered β-cell GCs to this process.

Macrophage infiltration in diabetic islets is an early event (1–4). Depletion (19) or inactivation (2) of macrophages prevents the progression of type 1 diabetes, highlighting the importance of the inflammatory mechanism *per se* in islet destruction. Reduction of macrophage infiltration in MIP-HSD1^tg/+^ islets is consistent with a reduced inflammatory insult that will work, at least in part, through longer-term curtailment of cytokine-mediated NO generation long after the STZ has been metabolized. GCs suppress a number of distinct pro-inflammatory signaling pathways that will also contribute to overall improvement in islet function (5). The higher Treg cell influx into MIP-HSD1^tg/+^ pancreas is consistent with increased islet GC levels influencing infiltrating macrophages to signal for resolution of inflammation (31). Increased T regulatory cell numbers also suggests that a greater suppression of pathogenic pro-inflammatory CD8^+^ Th1 cells and, intriguingly, may indicate enhanced suppression of self-antigens (32) and autoimmune responses over a longer time frame, which can only be addressed in future work in models such as the NOD mouse strain and in transplant models.

Of clinical significance, new technological advances in biohybrid β-cell transplant methods have achieved improvement of graft β-cell survival using slow-release of local corticosteroids, reportedly as a consequence of local immunosuppression (12). Our previous (14) and current data suggest that the secretory function and survival of encapsulated or transplanted human β-cells, which appear to express 11beta-HSD1 (33) could be potentially augmented through pre-treatment or local release of low-dose cortisone, thus, avoiding the deleterious effects of high dose or systemic GC administration that has hitherto precluded their use in this context.

## Author Contributions

Xiaoxia Liu and Sophie Turban designed and performed experiments. Roderick N. Carter, Shakil Ahmad, and Lynne Ramage performed experiments. Scott P. Webster, Brian R. Walker, and Jonathan R. Seckl commented on the manuscript and provided reagents. Nicholas M. Morton designed experiments and wrote the manuscript.

## Conflict of Interest Statement

Scott P. Webster, Brian R. Walker, Jonathan R. Seckl, and Nicholas M. Morton hold patents for the use of 11beta-HSD1 inhibitors for metabolic indications. The other coauthors declare that the research was conducted in the absence of any commercial or financial relationships that could be construed as a potential conflict of interest.

## Supplementary Material

The Supplementary Material for this article can be found online at http://www.frontiersin.org/Journal/10.3389/fendo.2014.00165/abstract

## References

[1] Eizirik DL Colli ML Ortis F The role of inflammation in insulitis and beta-cell loss in type 1 diabetes. Nat Rev Endocrinol (2009) 5:219–26 10.1038/nrendo.2009.21 19352320

[2] Hutchings P Rosen H O’Reilly L Simpson E Gordon S Cooke A Transfer of diabetes in mice prevented by blockade of adhesion-promoting receptor on macrophages. Nature (1990) 348:639–42 10.1038/348639a0 2250718

[3] Corbett JA Mikhael A Shimizu J Frederick K Misko TP McDaniel ML Nitric oxide production in islets from nonobese diabetic mice: aminoguanidine-sensitive and -resistant stages in the immunological diabetic process. Proc Natl Acad Sci U S A (1993) 90:8992–5 10.1073/pnas.90.19.8992 7692442 PMC47487

[4] Flodström M Tyrberg B Eizirik DL Sandler S Reduced sensitivity of inducible nitric oxide synthase-deficient mice to multiple low-dose streptozotocin-induced diabetes. Diabetes (1999) 48:706–13 10.2337/diabetes.48.4.706 10102685

[5] Silverman MN Sternberg EM Glucocorticoid regulation of inflammation and its functional correlates: from HPA axis to glucocorticoid receptor dysfunction. Ann N Y Acad Sci (2012) 261:55–63 10.1111/j.1749-6632.2012.06633.x 22823394 PMC3572859

[6] Shapiro AM Lakey JR Ryan EA Korbutt GS Toth E Warnock GL Islet transplantation in seven patients with type 1 diabetes mellitus using a glucocorticoid-free immunosuppressive regimen. N Engl J Med (2000) 343:230–8 10.1056/NEJM200007273430401 10911004

[7] Lambillotte C Gilon P Henquin JC Direct glucocorticoid inhibition of insulin secretion. An in vitro study of dexamethasone effects in mouse islets. J Clin Invest (1997) 99:414–23 10.1172/JCI119175 9022074 PMC507814

[8] Delaunay F Khan A Cintra A Davani B Ling ZC Andersson A Pancreatic beta cells are important targets for the diabetogenic effects of glucocorticoids. J Clin Invest (1997) 100:2094–8 10.1172/JCI119743 9329975 PMC508401

[9] Drachenberg CB Klassen DK Weir MR Wiland A Fink JC Bartlett ST Islet cell damage associated with tacrolimus and cyclosporine: morphological features in pancreas allograft biopsies and clinical correlation. Transplantation (1999) 68:396–402 10.1097/00007890-199908150-00012 10459544

[10] Hult M Ortsater H Schuster G Graedler F Beckers J Adamski J Short-term glucocorticoid treatment increases insulin secretion in islets derived from lean mice through multiple pathways and mechanisms. Mol Cell Endocrinol (2009) 301:109–16 10.1016/j.mce.2008.09.038 18984029

[11] Lund T Fosby B Korsgren O Scholz H Foss A Glucocorticoids reduce pro-inflammatory cytokines and tissue factor in vitro and improve function of transplanted human islets in vivo. Transpl Int (2008) 21:669–78 10.1111/j.1432-2277.2008.00664.x 18346012

[12] Buchwald P Bocca N Marzorati S Hochhaus G Bodor N Stabler C Feasibility of localized immunosuppression: 1. exploratory studies with glucocorticoids in a biohybrid device designed for cell transplantation. Pharmazie (2010) 65:421–8 20614690

[13] Rafacho A Marroqui L Taboga SR Abrantes JL Silveira LR Boschero AC Glucocorticoids in vivo induce both insulin hypersecretion and enhanced glucose sensitivity of stimulus-secretion coupling in isolated rat islets. Endocrinology (2010) 151:85–95 10.1210/en.2009-0704 19880808

[14] Turban S Liu X Ramage L Webster SP Walker BR Dunbar DR Optimal elevation of beta-cell 11beta-hydroxysteroid dehydrogenase type 1 is a compensatory mechanism that prevents high-fat diet-induced beta-cell failure. Diabetes (2012) 61:642–65 10.2337/db11-1054 22315313 PMC3282808

[15] Ehses JA Ellingsgaard H Böni-Schnetzler M Donath MY Pancreatic islet inflammation in type 2 diabetes: from alpha and beta cell compensation to dysfunction. Arch Physiol Biochem (2009) 115:240–7 10.1080/13813450903025879 19645635

[16] King A The use of animal models in diabetes research. Br J Pharmacol (2012) 166:877–94 10.1111/j.1476-5381.2012.01911.x 22352879 PMC3417415

[17] Kopp JL Dubois CL Schaffer AE Hao E Shih HP Seymour PA Sox9+ ductal cells are multipotent progenitors throughout development but do not produce new endocrine cells in the normal or injured adult pancreas. Development (2011) 138:653–65 10.1242/dev.056499 21266405 PMC3026412

[18] Xu X D’Hoker J Stange G Bonné S De Leu N Xiao X Beta cells can be generated from endogenous progenitors in injured adult mouse pancreas. Cell (2008) 132:197–207 10.1016/j.cell.2007.12.015 18243096

[19] Mensah-Brown E Shahin A Parekh K Hakim AA Shamisi MA Hsu DK Functional capacity of macrophages determines the induction of type 1 diabetes. Ann N Y Acad Sci (2006) 1084:49–57 10.1196/annals.1372.014 17151292

[20] Grinberg-Bleyer Y Saadoun D Baeyens A Billiard F Goldstein JD Grégoire S Pathogenic T cells have a paradoxical protective effect in murine autoimmune diabetes by boosting Tregs. J Clin Invest (2010) 120:4558–68 10.1172/JCI42945 21099113 PMC2993590

[21] Herold KC Bloch TN Vezys V Sun Q Diabetes induced with low doses of streptozotocin is mediated by V beta 8.2+ T-cells. Diabetes (1995) 44:354–9 10.2337/diabetes.44.3.354 7883124

[22] Elias D Prigozin H Polak N Rapoport M Lohse AW Cohen IR Autoimmune diabetes induced by the beta-cell toxin STZ. Immunity to the 60-kDa heat shock protein and to insulin. Diabetes (1994) 43:992–8 10.2337/diab.43.8.992 8039607

[23] Kwon NS Lee SH Choi CS Kho T Lee HS Nitric oxide generation from streptozotocin. FASEB J (1994) 8:529–33 8181671 10.1096/fasebj.8.8.8181671

[24] Reddy SI WU D Elliot RB Low dose streptozotocin causes diabetes in severe combined immunodeficient (SCID) mice without immune cell infiltration of the pancreatic islets. Autoimmunity (1995) 20:83–92 10.3109/08916939509001931 7578872

[25] Radomski MW Palmer RM Moncada S Glucocorticoids inhibit the expression of an inducible, but not the constitutive, nitric oxide synthase in vascular endothelial cells. Proc Natl Acad Sci U S A (1990) 87:10043–7 10.1073/pnas.87.24.10043 1702214 PMC55311

[26] Dor Y Brown J Martinez OI Melton DA Adult pancreatic beta-cells are formed by self-duplication rather than stem-cell differentiation. Nature (2004) 429:41–6 10.1038/nature02520 15129273

[27] Jonas JC Sharma A Hasenkamp W Ilkova H Patanè G Laybutt R Chronic hyperglycemia triggers loss of pancreatic beta cell differentiation in an animal model of diabetes. J Biol Chem (1999) 274:14112–21 10.1074/jbc.274.20.14112 10318828

[28] Yang J Zhang W Jiang W Sun X Han Y Ding M P21cip-overexpression in the mouse beta cells leads to the improved recovery from streptozotocin-induced diabetes. PLoS One (2009) 4(12):e8344 10.1371/journal.pone.0008344 20020058 PMC2792146

[29] Liu K Paterson AJ Chin E Kudlow JE Glucose stimulates protein modification by O-linked GlcNAc in pancreatic beta cells: linkage of O-linked GlcNAc to beta cell death. Proc Natl Acad Sci U S A (2000) 97:2820–5 10.1073/pnas.97.6.2820 10717000 PMC16013

[30] Kurrer MO Pakala SV Hanson HL Katz JD Beta cell apoptosis in T cell-mediated autoimmune diabetes. Proc Natl Acad Sci U S A (1997) 94:213–8 10.1073/pnas.94.1.213 8990188 PMC19288

[31] Kraaij MD van der Kooij SW Reinders ME Koekkoek K Rabelink TJ van Kooten C Dexamethasone increases ROS production and T cell suppressive capacity by anti-inflammatory macrophages. Mol Immunol (2011) 49:549–57 10.1016/j.molimm.2011.10.002 22047959

[32] Ono M Yaguchi H Ohkura N Kitabayashi I Nagamura Y Nomura T Foxp3 controls regulatory T-cell function by interacting with AML1/Runx1. Nature (2007) 446:685–9 10.1038/nature05673 17377532

[33] Schmid J Ludwig B Schally AV Steffen A Ziegler CG Block NL Modulation of pancreatic islets-stress axis by hypothalamic releasing hormones and 11beta-hydroxysteroid dehydrogenase. Proc Natl Acad Sci U S A (2011) 108:13722–7 10.1073/pnas.1110965108 21825133 PMC3158163

