# Supplementary figures and images for: β-Cell-Specific Glucocorticoid Reactivation Attenuates Inflammatory β-Cell Destruction
Source: Front Endocrinol (Lausanne). 2014 Oct 14;5:165. doi: 10.3389/fendo.2014.00165 (PMC4196588; doi:10.3389/fendo.2014.00165)

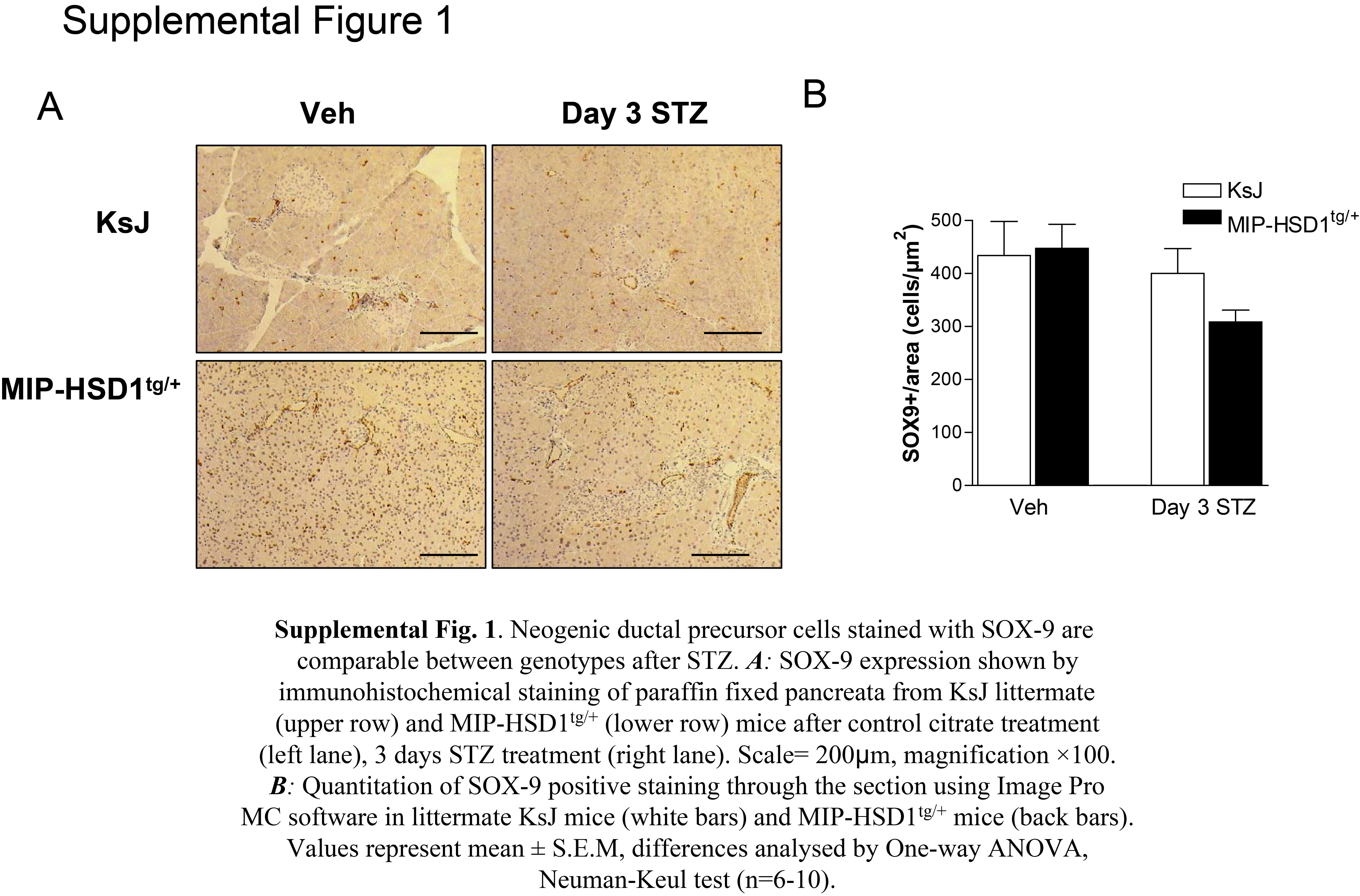

Supplement: Supplementary file 1 [file Image1.TIF]

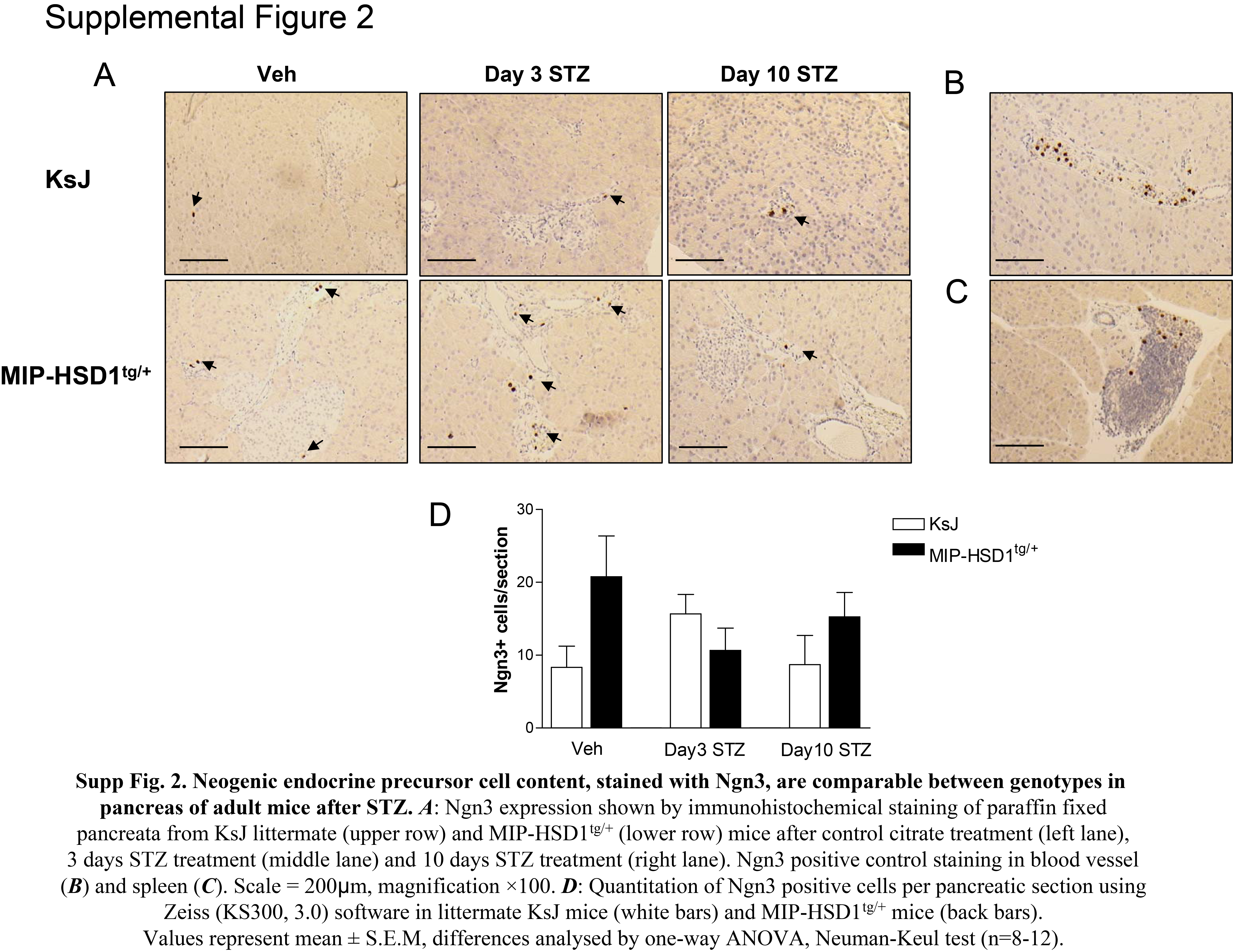

Supplement: Supplementary file 2 [file Image2.TIF]

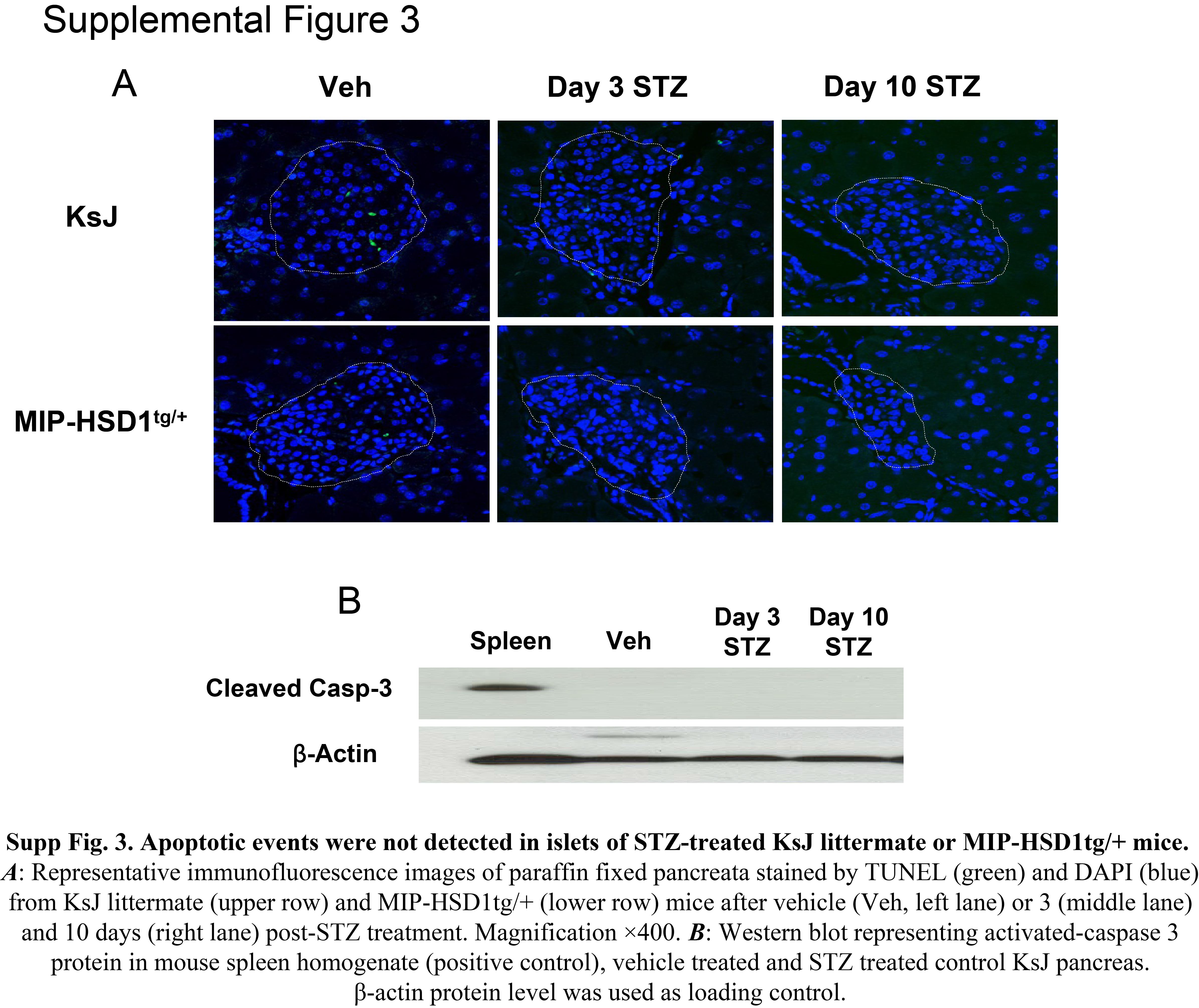

Supplement: Supplementary file 3 [file Image3.TIF]
